# Mindfulness, self-efficacy, and self-regulation as predictors of psychological well-being in EFL learners

**DOI:** 10.3389/fpsyg.2024.1332002

**Published:** 2024-03-27

**Authors:** Lijuan Fan, Feng Cui

**Affiliations:** ^1^College of Teacher Education, Weifang University of Science and Technology, Weifang, China; ^2^School of Marxism, Shandong University of Aeronautics, Binzhou, Shandong, China

**Keywords:** mindfulness, self-efficacy, self-regulation, psychological well-being, structural equation modeling, English as a foreign language (EFL)

## Abstract

**Introduction:**

Mindfulness, self-efficacy, and self-regulation play vital roles in shaping the psychological well-being of English as a Foreign Language (EFL) learners. This study investigates the interconnections among these constructs and their implications for the psychological well-being of 527 Chinese EFL learners.

**Methods:**

A cross-sectional study was conducted among Chinese EFL learners enrolled in a university in China. Participants were recruited through a non-probability convenience sampling method from English language courses. They completed validated self-report questionnaires assessing mindfulness, self-efficacy, self-regulation, and psychological well-being. Structural Equation Modeling (SEM) and mediation analysis were employed to explore the relationships among these constructs.

**Results:**

The study found that mindfulness and self-efficacy independently and directly predicted psychological well-being among Chinese EFL learners. Additionally, self-regulation emerged as a significant mediator in the relationship between mindfulness and psychological well-being, suggesting that mindfulness enhances well-being indirectly through improved self-regulation skills.

**Discussion:**

These findings underscore the critical roles of mindfulness practices, self-efficacy beliefs, and self-regulation skills in promoting psychological well-being among EFL learners. The implications of this study extend to mindfulness-based interventions and programs designed. However, the study’s cross-sectional design limits causal inference, and the use of self-report measures may introduce biases. Moreover, the sample’s limited diversity and homogeneous demographic profile, attributed to the convenience sampling from a single university, may constrain the generalizability of the findings. Future research could adopt longitudinal designs and diverse participant samples to further elucidate these relationships and enhance the robustness of the findings.

## Introduction

In the present fast-paced world characterized by mounting pressures, rapid technological advancements, and intricate societal changes, the pursuit of psychological well-being emerges as a paramount aspiration transcending various facets of life (Houben et al., 2015). Psychological well-being is more than just the absence of mental distress; it encompasses the presence of positive emotions, a sense of purpose, and a profound fulfillment in our everyday pursuits (Keyes, 2002; Anglim et al., 2020). Achieving and sustaining this state of well-being is a complex journey shaped by numerous individual and contextual factors (Claro and Perelmiter, 2022; Xiyun et al., 2022).

One such influential factor is self-efficacy, a foundational concept in social cognitive theory, as proposed by Bandura (1977). Self-efficacy revolves around an individual’s belief in their ability to not only execute tasks but also attain desired outcomes (Bandura, 1989). It stands as a potent predictor of motivation, performance, and persistence across a wide spectrum of domains, spanning from education and healthcare to personal development (Zimmerman, 2000; Livinƫi et al., 2021). Within the domain of psychological well-being, self-efficacy assumes a crucial role. Those possessing robust self-efficacy often approach challenges with resolute assurance, perceiving them as chances for personal development rather than formidable hurdles (Paciello et al., 2016; Phan, 2016).

Running parallel to self-efficacy is the concept of self-regulation, which encompasses the ability to monitor, control, and adapt one’s thoughts, emotions, and behaviors in alignment with personal goals and standards (Carver and Scheier, 1998; Zimmerman and Kitsantas, 2014). Proficient self-regulation not only propels the pursuit of long-term objectives but also maintains emotional equilibrium, both of which are intrinsic to psychological well-being (Hofer et al., 2011; Balkis and Duru, 2016; Fomina et al., 2020). Individuals armed with robust self-regulation skills are better equipped to navigate stress, surmount setbacks, and sustain a profound sense of purpose in their endeavors (Schunk, 1994; Gagnon et al., 2016).

Furthermore, the cultivation of psychological well-being is intricately associated with mindfulness, characterized by a state of non-judgmental awareness of the present moment (Brown and Ryan, 2003). Mindfulness practices have demonstrated a capacity to reduce stress, enhance emotional regulation, and elevate life satisfaction (Hofmann et al., 2010; Tang et al., 2015; Goretzki and Zysk, 2017). Nevertheless, the potential interplay between self-efficacy, self-regulation, and mindfulness, and how these constructs collectively shape psychological well-being, remains an evolving area of inquiry.

To bridge this critical gap in the existing body of knowledge, this study embarks on an exploration of the interrelationships between self-efficacy, self-regulation, mindfulness, and psychological well-being. Through a comprehensive examination of these dynamic interactions, we aspire to offer nuanced insights that can inform targeted interventions and strategies aimed at fortifying psychological well-being within various contexts, ranging from educational settings to clinical practice and beyond.

The Chinese context might present a unique set of cultural and educational factors that can influence the interplay between self-efficacy, self-regulation, mindfulness, and psychological well-being in EFL learners. For instance, Chinese culture emphasizes collective goals and conformity, which may impact individuals’ self-efficacy perceptions and self-regulation strategies (Zhu and Li, 2019). Additionally, the competitive nature of Chinese education can place significant pressure on learners, potentially affecting their psychological well-being (Zhao, 2015).

The findings of this study might have important implications for language education, suggesting that enhancing self-efficacy, self-regulation, and mindfulness in EFL learners can promote their psychological well-being and overall language learning success. Language teachers can incorporate mindfulness-based interventions, self-efficacy-building activities, and self-regulation strategies into their instruction to foster a supportive and empowering learning environment. These interventions can help EFL learners manage stress, improve focus, and develop a growth mindset, which can positively impact their language learning experiences.

Specifically, mindfulness practices might help EFL learners become more aware of their thoughts and emotions, enabling them to better manage negative emotions and cultivate a sense of equanimity. Self-efficacy-building activities may instill in learners a belief in their ability to succeed in language learning, encouraging them to persist in the face of challenges. Self-regulation strategies can also equip learners with the skills to manage their time effectively, set realistic goals, and overcome procrastination, leading to improved language proficiency and overall well-being. By incorporating mindfulness, self-efficacy, and self-regulation into language instruction, educators can empower EFL learners to develop the resilience, adaptability, and emotional regulation skills necessary to thrive in both their language learning journeys and broader life experiences.

## The literature review

### Psychological well-being

The concept of psychological well-being is multifaceted and has been approached from various theoretical perspectives within the field of psychology. One prominent theoretical tradition, rooted in hedonism or the subjective well-being approach, characterizes well-being as synonymous with pleasure or happiness (Kahneman, 1999). This viewpoint encompasses three core components: contentment with life, the existence of favorable emotions, and the lack of adverse emotions, collectively termed as ‘happiness’ (Ryan and Deci, 2001). Empirical psychology has placed considerable emphasis on subjective well-being due to its associations with both physical and mental health. Factors such as life satisfaction, positive mood, hope, and happiness have been linked to various mental disorders (Seligman et al., 2006). This viewpoint underscores the importance of mental and physical preferences and pleasures (Zhang et al., 2022).

In contrast to the subjective well-being approach, an alternative perspective, termed the eudaimonic tradition, posits that psychological well-being centers on the acknowledgment and realization of an individual’s innate potential (Ryff and Singer, 2008). This standpoint underscores an individual’s feeling of self-governance, the significance and worth of their endeavors, and the caliber of their social connections. This theory is commonly denoted as eudaimonism or psychological well-being, underscoring the idea that well-being is attained through the expression of one’s daimon or authentic essence (Houben et al., 2015).

While the eudaimonic approach was somewhat overshadowed by the subjective well-being perspective for an extended period (Wood and Joseph, 2010), it has recently garnered empirical attention and become a focal point in clinical treatments (Casale et al., 2015). This shift is of considerable significance because psychological well-being and subjective well-being explore distinct facets of the human experience. In this study, psychological well-being is defined and assessed using the eudaimonic tradition, emphasizing an individual’s sense of purpose in life, personal growth, and the quality of their relationships with others (Choi and Kim, 2016). This perspective delves into the profound aspects of human potential and fulfillment, offering an additional lens through which to understand the complex phenomenon of psychological well-being. It is this nuanced approach that guides our exploration of self-efficacy within the context of psychological well-being, where individuals’ beliefs about their capabilities become central in the pursuit of a fulfilling and purpose-driven life.

Psychological well-being, a multi-dimensional construct, encompasses an individual’s ability to not only experience positive emotions and life satisfaction but also effectively navigate and cope with negative emotions, recognizing them as an integral part of the human experience (Diener, 2006). It is essential to emphasize that the assessment of psychological well-being relies on subjective judgments rather than objective criteria, taking into account an individual’s emotional history, memories, aspirations, values, and benchmarks for comparison (Biswas-Diener et al., 2004).

Cognitive elements also play a central role in shaping an individual’s sense of well-being. Kafka and Kozma (2002) highlight that people tend to experience greater contentment when they perceive advancement within a context of their own choosing. Ryff (1989) foundational work further delineates the constituents of psychological well-being, covering autonomy, mastery over one’s surroundings, the development of positive relationships, the pursuit of a meaningful life, personal growth, and self-acceptance. Extending upon this groundwork, Keyes (1998) advocates for the inclusion of the communal dimension within this framework, underscoring the significance of social connections in overall well-being. Seligman (2011) broadens this discussion by suggesting that psychological well-being possesses a multidimensional structure. He argues that it encompasses positive emotions, attachment, nurturing relationships, the quest for meaning, and a sense of achievement. In essence, psychological well-being goes beyond the absence of pathology, encapsulating a comprehensive assessment of one’s life, experiences, body, mind, and surrounding circumstances (Diener, 2006; Fathi and Mohammaddokht, 2021).

This comprehensive understanding of psychological well-being sets the stage for our exploration of the predictors of well-being, including mindfulness, self-efficacy, and self-regulation. It highlights the need for a holistic approach that considers the interplay of cognitive, emotional, and relational factors in shaping individuals’ psychological well-being.

### Mindfulness

Mindfulness, stemming from ancient spiritual traditions, has received considerable attention for its profound impact on behavior and overall well-being (Baer et al., 2004; Crane, 2017). It entails a deliberate state of presence achieved by non-judgmentally focusing on one’s objectives and fully immersing oneself in the present moment (Brown and Ryan, 2003; Boyce, 2011). This quality is especially pertinent in the realm of psychological well-being, as excessive fixation on past memories and future possibilities can lead to increased anxiety and apprehension. Recent research has explored the intricate relationship between mindfulness and various psychological factors, including neuroticism, depression, and psychological distress (Armstrong and Rimes, 2016).

Mindfulness has emerged as a pivotal element in understanding and alleviating anxiety and anxiety-related disorders (Bamber and Schneider, 2016). It has proven effective in reducing both the physical and mental symptoms of anxiety. Moreover, mindfulness has shown its potential to help individuals confront factors that could threaten their performance and well-being. For example, study in Thailand highlighted the positive correlation between mindfulness and improvisational behavior in entrepreneurs, especially during challenging periods. Similarly, Charoensukmongkol and Puyod (2022) demonstrated mindfulness’s ability to reduce emotional exhaustion among high-demand call center agents. These findings underscore the value of mindfulness training as a proactive strategy for individuals and organizations to mitigate the adverse consequences of stress and enhance overall well-being.

The evolution of mindfulness research has led to a broadening of its scope beyond its traditional Buddhist roots. Kabat-Zinn (2003), a pioneer in clinical applications, eloquently characterizes mindfulness as the awareness that arises from a deliberate, nonjudgmental focus on the unfolding of present moment experiences. Duan (2014), adopting a positive psychology perspective, views mindfulness as a trait that combines inherent and learned elements, closely linked to characteristics like inquisitiveness, receptivity, and an open-minded approach to the present moment (Kabat-Zinn, 2003).

Mindfulness research began with a focus on its applications in clinical medicine, where mindfulness-based interventions were found to significantly amplify positive emotions and mitigate negative ones, particularly in mood regulation and depression treatment (Teasdale et al., 2000). Beyond clinical settings, scholars unveiled mindfulness’s potential in enhancing metacognition (Shapiro et al., 2006), promoting individual well-being (Brown and Ryan, 2003), and moderating negative emotions (Coffey et al., 2010). The integration of positive psychology into the field of education prompted an in-depth examination of the influence of mindfulness on academic emotions, revealing its role as a predictor and regulator of test anxiety (Wang and Zhao, 2015), a method for ameliorating attention and academic emotions in students with learning disabilities (Chen et al., 2019), and a tool for enhancing students’ resilience against boredom and improving their commitment to academic endeavors (Galla et al., 2020; Mohammad Hosseini et al., 2023).

In an extensive systematic review, Lomas et al. (2017) confirmed the positive outcomes of mindfulness on individual well-being and workplace performance. Hoffman (2010) further substantiated this idea by emphasizing the potential enhancement of overall well-being through mindfulness exercises. Klussman et al. (2020) delved into the underlying mechanisms, suggesting that self-connection plays a pivotal role as an intermediary in the relationship between mindfulness and well-being. Research by Zollars et al. (2019) explored the effects of mindfulness meditation, finding it to make significant contributions to heightened mindfulness, mental well-being, and a reduction in perceived stress. Goretzki and Zysk (2017) extended the perspective to university students, demonstrating the potential of mindfulness techniques to bolster both student welfare and academic performance.

These collective studies underscore the consistent and multifaceted advantages of integrating mindfulness practices across a range of domains, ultimately leading to improvements in individuals’ overall well-being. The relevance of mindfulness to learners’ psychological well-being, within the context of our research, is well-supported by this extensive body of literature.

### Academic self-efficacy

According to Bandura’s Social Cognitive Theory (Bandura, 1989), individuals are not passive recipients of external influences nor purely independent actors. They actively shape their motivation and behaviors within a system of reciprocal causation, which comprises actions, the environment, and personal factors, including self-efficacy beliefs. Self-efficacy, as defined by Bandura (1997), denotes one’s confidence in effectively planning and executing actions needed to achieve specific goals. It reflects an individual’s assurance in autonomously navigating complex situations and surmounting various challenges.

Within this framework of reciprocal causation, self-efficacy plays a pivotal role as a personal factor influencing human behavior through cognitive, motivational, and emotional processes (Bandura, 1989). It shapes human conduct by fostering constructive thought patterns, driving the exertion of effort, and influencing the experience of stress. Contextual and domain-specific factors are crucial considerations when assessing self-efficacy, as individuals’ self-assessments of their capabilities can markedly vary under different circumstances (Bandura and Locke, 2003).

In an educational context, academic self-efficacy epitomizes individuals’ belief in their ability to confidently navigate their educational journey. This concept centers on the expectation of competence and the capability to adeptly handle the academic challenges inherent in educational environments. Importantly, academic self-efficacy is closely interlinked with broader dimensions of self-efficacy and exhibits a substantial positive correlation with overall self-confidence, optimism, and social self-efficacy, which pertains to an individual’s perceived capacity to initiate and sustain social interactions (Artino, 2012; Galla et al., 2014).

Academic self-efficacy forms the cornerstone of an individual’s belief in their capacity to excel academically. It profoundly influences learners’ approach to educational challenges and their persistence in academic endeavors. The intricate interaction between self-efficacy and academic achievement stands at the core of the educational landscape. When students possess a strong sense of academic self-efficacy, they view challenges as manageable stressors that can be surmounted, which, in turn, propels them toward persistent efforts to achieve their goals (Bandura, 1986; Schunk and Pajares, 2005). Attaining academic success, in return, elicits a pleasant affective response to learning, enhancing innate motivation and the perceived value of tasks. This sets in motion a positive feedback loop because individuals with greater scholarly efficacy perceptions tend to persist when confronted with adversity, ultimately reaching their goals and further reinforcing their academic self-efficacy (Lee et al., 2014). Conversely, students possessing diminished academic self-assurance could exhibit adverse reactions when confronted with scholastic difficulties, potentially succumbing more easily to stressors. This, in turn, reinforces their unfavorable self-view, potentially culminating in a condition of acquired helplessness (Stipek, 1988).

In the sphere of education, this materializes as academic self-efficacy, particularly in the context of English language acquisition. Here, learners assess their proficiency in accomplishing academic tasks and meeting the demands of English language learning (Huang, 2013). The relevance of domain-specific self-efficacy beliefs becomes apparent, particularly in the arena of English language acquisition.

In China, there is a growing body of research dedicated to exploring self-efficacy in English learning, with a specific focus on college students, particularly those not majoring in English. However, there remains a dearth of research data concerning learners (Mo, 2012). Studies centered on middle school students primarily delve into the intricate interplay between self-efficacy in English learning and various educational facets, including learning motivation, emotional experiences in learning (Wang, 2017), and academic performance (Peng, 2018).

The concept of self-efficacy theory emphasizes that an individual’s belief in their capabilities is not fixed but can be influenced by their prior achievements and setbacks (Bandura, 1989). Strong adaptability to learning environments suggests successful adjustment to these settings, ultimately boosting one’s self-assurance. Previous research consistently illustrates a robust positive relationship between learning adaptability and the academic self-efficacy of middle school students (Zhang, 2012). Those with high self-efficacy not only possess confidence in their skills but also offer more favorable self-evaluations. Consequently, they invest more effort and cognitive resources when confronted with challenging tasks. On the flip side, those with diminished self-efficacy often gravitate toward avoidance tactics. As a result, academic self-efficacy demonstrates a strong interconnection with academic participation (Schunk and Pajares, 2005). Specifically, in the context of middle school students, their academic self-efficacy can be seen as a harbinger of their active involvement in the learning process (Li and Bai, 2018).

The amalgamation of these research endeavors collectively emphasizes the substantial connection between self-efficacy and overall wellness, with a particular focus on academic contexts. Phan’s (2016) longitudinal investigation unveiled a pathway in which optimism and personal self-efficacy positively affect the wellbeing of students. Siddiqui (2015) concentrated on the undergraduate demographic, illustrating the influence of self-efficacy on psychological welfare, underscoring its pertinence in the academic sphere.

Etherton et al. (2022) extended this perspective by delving into the role of resilience in student performance and holistic wellness, accentuating the pivotal function of self-efficacy, self-determined objectives, and the amelioration of anxiety in attaining favorable outcomes. Paciello et al. (2016) took a person-centric approach, pinpointing discrete self-efficacy configurations that correspond with varying degrees of wellbeing within the academic milieu.

In a longitudinal study, Cobo-Rendón et al. (2020) underscored the interwoven nature of affective wellbeing, psychological welfare, self-efficacy, and academic achievement among first-year undergraduate students. In summation, these studies collectively offer persuasive substantiation for the indispensable role of self-efficacy in augmenting wellbeing, encompassing both the mental and educational dimensions, and its relevance throughout diverse phases of the educational journey.

### Self-regulation

Self-regulation (SRL) stands as a foundational concept in educational psychology, particularly salient in the realm of second language acquisition (Zheng et al., 2018; Jansen et al., 2019). This intricate and dynamic construct forms the cornerstone of learner autonomy and self-determination (Dornyei and Ryan, 2015). In the context of second language acquisition, SRL empowers language learners to take command of their cognitive, emotional, and behavioral facets, aligning them with their educational objectives (Zimmerman and Kitsantas, 2014). This transformative process facilitates the development of language proficiency, effectively bridging the divide between a learner’s cognitive capacities and their linguistic aptitude (Dörnyei, 2005). The concept of self-regulation encompasses various interconnected processes, including goal-setting, progress monitoring, emotional regulation, and the utilization of strategic learning techniques, collectively spanning the entire language learning journey (Sitzmann and Ely, 2011). Learners are tasked with proactively determining what and how they learn, sustaining motivation by managing emotional responses, and adjusting strategies as they progress (Theobald, 2021). Self-regulation not only impacts linguistic proficiency but also profoundly shapes the overall learning experience (Kim et al., 2015). Learners adept at self-regulation are better equipped to overcome challenges and persevere in their language learning pursuits, ultimately leading to greater success in acquiring a foreign language (Teng and Zhang, 2016; Lei et al., 2022).

Additionally, self-regulated learning plays a pivotal role in learners’ academic advancement, rooted in social cognitive theory. This theory posits that self-regulated learning arises from the dynamic interplay between personal attributes, behaviors, and environmental factors (Schunk, 2001). At the core of a self-regulated learner’s belief system lies the concept of academic self-efficacy, as proposed by Bandura and Locke (2003). Academic self-efficacy, the confidence in one’s ability to excel academically, forms the bedrock of one’s sense of agency and serves as a key motivator in academic pursuits (Zimmerman, 2000).

From a cognitive standpoint, belief in one’s abilities shapes the selection of academic activities and encourages a thoughtful approach to academic challenges (Panadero et al., 2017). This belief also fosters strategic attributions tied to one’s sense of control. The understanding that current abilities can improve through effort is widely acknowledged as the most psychologically adaptable viewpoint (Weiner, 2005). It equips students with the understanding that they can master any subject matter by employing effective strategies, thereby promoting academic persistence and accomplishment.

From a motivational perspective, individuals characterized by elevated academic self-confidence frequently establish more challenging educational aspirations (Zimmerman and Kitsantas, 2014). The Achievement Goal Theory delineates two constructive learning goal classifications: mastery approach and performance approach. The former seeks to elevate competence, while the latter aims to showcase competence (Elliot, 2005). Research indicates that a combination of high mastery and high performance approach goal orientations may be the most psychologically adaptable, as they are associated with intrinsic interest and greater achievement (Harackiewicz et al., 2002).

The amalgamation of these research endeavors collectively highlights the complex interrelationship between self-regulation and overall well-being, with diverse studies shedding light on various facets of this association. Fomina et al. (2020) undertook a longitudinal study spanning two waves, offering insights into the involvement of self-regulation during early adolescence and its impact on psychological health. Mascia et al. (2020) delved into the interplay between emotional intelligence, self-regulation, and smartphone addiction, exploring their influence on the well-being and quality of life of students. This research delved into how self-regulation competencies are intertwined with emotional intelligence and, consequently, contribute to the students’ overall well-being.

In another study, Gagnon et al. (2016) delved into the significance of self-regulation within the sphere of medical practitioners and medical students, uncovering its connection to both well-being and the risk of burnout. This study underlines the necessity of nurturing self-help skills among these professionals. Balkis and Duru (2016) delved into the consequences of procrastination and self-regulation lapses on academic life satisfaction and emotional well-being, distinguishing between under-regulation and misregulation modes of self-regulation. Also, Hofer et al. (2011) considered the impact of identity and motives on self-regulation and overall well-being. Their research underscores the intricate link between self-regulation, one’s sense of identity, and the underlying motivations, further influencing one’s overall state of well-being. Overall, these studies collectively underscore the multifaceted role of self-regulation in shaping well-being, spanning various phases of life, occupational domains, and emotional dimensions.

### Research hypotheses

*H1*: Mindfulness is directly related with psychological well-being

Mindfulness practices, encompassing meditation, deep breathing exercises, and awareness of present experiences, have been consistently associated with positive psychological outcomes. Existing literature supports the idea that engaging in mindfulness is directly linked to improved psychological well-being. Studies demonstrate that individuals practicing mindfulness exhibit lower stress levels (Khoury et al., 2015), reduced symptoms of anxiety and depression (Hofmann et al., 2010), and increased life satisfaction (Brown and Ryan, 2003). Moreover, the literature suggests that mindfulness is connected to enhanced emotional regulation and cognitive functioning, ultimately contributing to improved psychological well-being in areas such as self-awareness, emotional resilience, and cognitive clarity (Hoffman, 2010; Ager et al., 2015; Goretzki and Zysk, 2017; Lomas et al., 2017; Zollars et al., 2019; Klussman et al., 2020). Therefore, H1 is well-justified based on the existing literature.

*H2*: Self-efficacy is directly associated with psychological well-being

The hypothesis posits that high self-efficacy is directly linked to favorable psychological outcomes. Extending this, individuals with greater self-efficacy are expected to exhibit higher self-esteem, reduced stress levels, and an overall sense of well-being. The literature consistently supports this hypothesis, highlighting that individuals with high self-efficacy experience a heightened sense of mastery and control, contributing significantly to their psychological well-being (Siddiqui, 2015; Paciello et al., 2016). This association has been well-documented across various studies in psychology, reinforcing the validity of H2 (Siddiqui, 2015; Paciello et al., 2016; Phan, 2016; Cobo-Rendón et al., 2020; Etherton et al., 2022).

*H3*: Self-regulation mediates the relationship between mindfulness and psychological well-being

Mindfulness practices inherently involve specific self-regulatory processes such as attention control and emotional regulation. Engaging in mindfulness is expected to cultivate enhanced self-regulation skills, contributing to heightened psychological well-being. The mediation hypothesis (H3) aligns with prior research indicating that self-regulation serves as a mediator in the relationship between mindfulness and psychological well-being (Chambers et al., 2008; Lundwall et al., 2019; Fathi et al., 2023). To provide further precision, attention control is specified as the cognitive dimension, and emotional regulation as the affective dimension of self-regulation in the mediation process.

*H4*: Self-regulation mediates the relationship between self-efficacy and psychological well-being

The interconnection between self-efficacy and self-regulation is well-documented. Individuals with elevated self-efficacy levels frequently exhibit enhanced self-regulation skills. These self-efficacious individuals are more inclined to engage in proactive and adaptive self-regulation behaviors, leading to improved psychological well-being. High self-efficacy is expected to drive individuals to set and diligently pursue their goals, while effective self-regulation strategies empower them to attain these objectives. Existing studies underscore that self-regulation acts as a mediator in the relationship between self-efficacy and psychological well-being. To provide further specificity, goal-setting, monitoring progress, and adaptive strategies are highlighted as dimensions of effective self-regulation in reinforcing the validity of H4 (Schunk, 1994; Paciello et al., 2016; Phan, 2016; Cobo-Rendón et al., 2020; Etherton et al., 2022).

## Materials and methods

### Participants

The participants consisted of 527 intermediate EFL learners, hailing from a diverse array of educational institutions situated in the northern and eastern regions of China. The participants were drawn from urban and rural settings, encompassing a spectrum of academic environments such as universities, secondary schools, and language training centers. This geographic diversity was strategically designed to enrich the generalizability of the study findings beyond specific institutional contexts. For instance, participants from urban areas might be exposed to different educational resources and socio-cultural influences compared to their counterparts in rural settings, contributing to a more nuanced understanding of the impact of mindfulness, self-efficacy, and self-regulation on psychological well-being.

The participant distribution was meticulously balanced in terms of gender, with 246 male participants, constituting 46.7% of the total cohort, and 281 female participants, representing 53.3% of the sample. This gender equilibrium sought to minimize potential gender-related biases in the study’s outcomes. The participants’ ages ranged from 18 to 30 years old, with an average age of 21.93 (SD = 2.14). This age range is representative of the typical age group of intermediate EFL learners in China. Educational background was another important demographic factor taken into account. The participants were enrolled in various educational institutions, including universities, vocational schools, and language institutes. The diverse educational backgrounds aimed to encompass a variety of learning environments and experiences, contributing to the generalizability of the study findings across different educational settings.

Selection of participants was methodically carried out to ensure a uniform and consistent level of English proficiency across the cohort. All participants were required to possess an intermediate level of competence in the English language, which was determined through standardized language proficiency assessments and validated placement tests. This rigorous selection criterion guaranteed that participants shared a common baseline level of language skills.

To assemble this diverse yet academically homogeneous cohort, a convenience sampling method was employed. This method was chosen for its practicality in accessing a broad spectrum of intermediate EFL learners while maintaining the desired level of English proficiency within the scope.

### Measures

#### Academic self-efficacy

To assess academic self-efficacy, the subscale derived from the Motivated Strategies for Learning Questionnaire (MSLQ; Pintrich and De Groot, 1990) was employed. This subscale, consisting of items such as “I expect to do well in this class,” was carefully translated into Chinese and subsequently back-translated into English to ensure cultural equivalence. The average item score was computed and standardized to represent academic self-efficacy within the model.

The MSLQ subscale was used as an appropriate measure of academic self-efficacy given its established psychometric properties and its alignment with the theoretical framework of the study. This subscale has demonstrated strong internal consistency (α = 0.87) and convergent validity with other measures of self-efficacy (Pintrich and De Groot, 1990).

#### Mindfulness

The Mindful Attention Awareness Scale (MAAS; Brown and Ryan, 2003) was employed to assess mindfulness. This 15-item instrument, featuring statements such as “I often work automatically on tasks without being aware of what I am doing,” utilizes a six-point Likert scale ranging from most always (1) to almost never (6). The MAAS has exhibited robust internal consistency (α = 0.96) and excellent convergent and discriminant validity (Bajaj et al., 2016).

The MAAS was employed due to its established psychometric properties and its strong alignment with the conceptualization of mindfulness as non-judgmental awareness of the present moment. The MAAS has been widely used in empirical research and has demonstrated its ability to capture the essence of mindfulness among Chinese adolescents (Black et al., 2012).

#### Self-regulation

The Short Self-Regulation Questionnaire (SSRQ; Carey et al., 2004), comprising 31 items such as “I usually keep track of my progress toward my goals,” was employed to assess self-regulation. Participants rated their agreement on a five-point Likert scale ranging from 1 (strongly disagree) to 5 (strongly agree). The SSRQ exhibited high internal consistency in the current study, with a Cronbach’s alpha coefficient of 0.92. The SSRQ was chosen due to its comprehensive assessment of various dimensions of self-regulation, including goal setting, planning, and perseverance. The SSRQ has demonstrated adequate reliability and validity in previous research in Chinese settings (e.g., Chen and Lin, 2018).

#### Psychological well-being

To gauge participants’ psychological well-being, the study employed the Psychological Well-Being Scale, developed by Ryff and Keyes (1995). This instrument consists of 18 items, each designed to measure various dimensions of well-being, including favorable interpersonal connections, individual development, self-acknowledgment, a sense of purpose, self-determination, and adeptness in handling one’s surroundings. Participants rated their agreement using a scale ranging from strongly disagreed (1) to strongly agreed (7). The scale holds a firm and established position in the academic corpus, having been extensively employed in prior research endeavors (Ryff and Keyes, 1995).

The PWB was chosen due to its comprehensive assessment of the multidimensional nature of psychological well-being. The PWB has demonstrated strong psychometric properties, including high internal consistency and convergent and discriminant validity in Chinese context (Gao and McLellan, 2018).

#### Data collection procedure

The recruitment process for this study was meticulously planned in collaboration with educational institutions situated in the northern and eastern regions of China. Formal informed consent was obtained from these institutions, granting permission to access their students for research purposes. In alignment with institutional protocols, participants were approached with utmost transparency and clarity regarding their participation in the study.

Before the initiation of data collection, each participant received a brief pre-survey questionnaire. This questionnaire assessed participants’ familiarity with the study’s constructs, identifying any areas of ambiguity or misinterpretation. The insights gained from this questionnaire prompted additional clarification during the informed consent process, ensuring a comprehensive understanding of the study.

Upon completion of the study, participants were thanked sincerely for their valuable contributions, and gratitude was expressed for their active participation. To further recognize their involvement, participants were offered research participation credit, contributing toward their course requirements and providing them with a tangible benefit.

In addition to the tangible benefits, participants were reminded of the opportunity for self-reflection during the study. Engaging in the survey allowed participants to reflect on their mindfulness, self-efficacy, self-regulation, and psychological well-being. This opportunity for self-reflection was emphasized as a potential intrinsic benefit of participation.

To facilitate data collection, a well-structured questionnaire was administered through a secure online platform. Each participant received a unique access link, a crucial measure to maintain the confidentiality and security of their responses. The online platform provided participants with the flexibility to complete the survey at their convenience, ensuring that they could allocate the necessary time without feeling undue pressure. The estimated duration for survey completion typically ranged from 20 to 30 min.

In accordance with ethical standards, stringent safeguards were implemented to protect the anonymity and confidentiality of each participant. Personal information was kept distinctly separated from the collected data, and all responses were securely stored. Access to the data repository was restricted exclusively to authorized members of the research team, reinforcing the protection of participants’ sensitive information.

#### Ethical considerations

Pursuant to the ethical principles of research, the present study adhered to the highest standards of conduct and adhered to the ethical guidelines set forth by the College of Teacher Education, Weifang University of Science and Technology. Prior to commencing data collection, the study protocol was meticulously reviewed and approved by the College’s Institutional Review Board (IRB). This approval ensured that the study’s methodology and procedures aligned with ethical principles and safeguarded the well-being of participants.

To protect the privacy and anonymity of participants, informed consent forms were obtained from all participants. These forms clearly outlined the study’s objectives, procedures, potential risks and benefits, and the voluntary nature of participation. Participants were informed that they were free to withdraw from the study at any point without penalty.

Confidentiality was paramount, and all personal information collected from participants was handled with the utmost care. Participants’ responses were assigned unique identifiers to maintain their anonymity, and data was stored securely in password-protected electronic files. Also, the research team emphasized transparency and open communication throughout the study. Participants were kept informed of the progress of the study, and their feedback was actively sought and incorporated into the research process.

#### Analytic approach

The data analysis procedure in this study encompassed a series of methodical steps designed to explore the interrelationships among the variables and rigorously test the research hypotheses. To this end, a comprehensive analytical framework was employed. Initial analyses involved descriptive and correlation assessments, executed through SPSS version 28.0, aimed at illuminating the characteristics of the variables and elucidating their associations.

Structural Equation Modeling (SEM) was employed with the use of the Amos program (version 26.0) as the primary analytical method for several reasons. First, SEM is a powerful statistical tool that allows for the simultaneous assessment of complex relationships among multiple variables. This capability was essential for our study, as we sought to investigate the intricate interplay between mindfulness, self-efficacy, self-regulation, and psychological well-being.

Second, SEM enables the examination of both direct and indirect effects, providing a more comprehensive understanding of the mechanisms underlying the observed associations. This feature was crucial for our research, as we hypothesized that mindfulness indirectly influenced psychological well-being through self-efficacy and self-regulation. SEM allowed us to quantify these indirect effects, providing a deeper insight into the mediating role of these variables.

Third, SEM offers a rigorous framework for evaluating the overall fit of the proposed model to the data. This assessment is crucial for ensuring that the model accurately represents the underlying relationships among the variables. An array of fit indices was strategically employed to gauge the overall adequacy of the proposed model. Central to this assessment was the χ^2^-goodness of fit to degree of freedom (df) ratio, where a value below 3 was indicative of a satisfactory fit. Additionally, the Goodness of Fit Index (GFI) and the Comparative Fit Index (CFI) played pivotal roles in the evaluation. A GFI and CFI value equal to or greater than 0.90 was considered indicative of a well-fitting model.

Further scrutiny was undertaken through the evaluation of the Root-Mean-Square Error of Approximation (RMSEA) and the Standardized Root-Mean-Square Residual (SRMR) as additional indicators of model fit. Conventionally, an RMSEA value below 0.08 and an SRMR value under 0.10 were regarded as robust evidence of a model that fits well with the data, in alignment with established criteria (Huang and Bentler, 1999).

## Results

In the initial phase of our analysis, we conducted preliminary assessments to ensure data quality and adherence to assumptions before proceeding with the proposed model. These critical preliminary analyses were carried out using SPSS version 28, encompassing a thorough examination of missing data, normality, and potential outliers, in line with established practices (Tabachnick and Fidell, 2007).

Addressing missing data is a crucial step in data preparation. In cases of substantial missing data, methods like list-wise deletion or pair-wise deletion are often impractical, particularly when dealing with smaller sample sizes. Given the context of our study and the prevalence of missing data, we opted for the Expectation–Maximization (EM) algorithm, an effective imputation technique that replaces missing data points with estimated values (Kline, 2015). Subsequently, we examined the normality of our data by assessing skewness and kurtosis indices for each item. A skewness or kurtosis value exceeding ±2.0 is indicative of a non-normal distribution. Items displaying such characteristics were meticulously identified and subsequently removed from our dataset.

Furthermore, we conducted a comprehensive evaluation to detect univariate and multivariate outliers. To identify univariate outliers, *Z*-standardized scores were employed, and for multivariate outliers, the Mahalanobis *D*^2^ measure was applied, all in accordance with established guidelines (Tabachnick and Fidell, 2007). Outliers, once identified, were systematically removed from the dataset.

In our pursuit of rigorous data analysis, we embarked on a comprehensive examination of construct validity following the initial data screening procedures. To assess the suitability of our measurement models, we employed Confirmatory Factor Analysis (CFA) and scrutinized the performance of these models using various goodness-of-fit indices (Hair et al., 1998). This process involved the evaluation of measurement models for all latent constructs, including those yet to be mentioned.

It is noteworthy that a subset of the initial measurement models did not exhibit a satisfactory fit with our dataset. To rectify this, deliberate model adjustments were undertaken to enhance their alignment with the empirical data. Specifically, this optimization process involved the removal of three items from our scales, which showed factor loadings falling below the recommended threshold of 0.40. Additionally, we introduced two correlational paths between error terms associated with two latent constructs, thereby refining the models.

Following these strategic modifications, the final measurement models emerged with a commendable level of fit, as comprehensively detailed in Table 1. This signifies the successful alignment of our measurement models with the empirical data, reaffirming their efficacy in capturing the underlying constructs.

**Table 1:** The outcome of measurement model.

	_χ_2	df	χ^2^/df	CFI	TLI	RMSEA
Mindfulness	87.12	43	2.02	0.94	0.93	0.05
Self-efficacy	104.13	54	1.92	0.95	0.94	0.04
Self-regulation	56.22	31	1.81	0.97	0.97	0.04
Well-being	338.24	204	1.65	0.98	0.97	0.03

Moreover, to extend our understanding of the factors under investigation, we conducted an exploration of the descriptive statistics and correlations for all variables, a summary of which is thoughtfully presented in Table 2. Descriptive statistics revealed that the mean score for mindfulness was 3.42 (SD = 0.62). For self-efficacy, the mean score was 2.94 (SD = 0.56), and for self-regulation, the mean score was 3.16 (SD = 0.85). Additionally, the mean score for psychological well-being was 4.03 (SD = 0.64).

**Table 2:** Descriptive statistics.

	*M* (*SD*)	1	2	3	4
1. Mindfulness	3.42 (0.62)	1.00			
2. Self-efficacy	2.94 (0.56)	0.23*	1.00		
3. Self-regulation	3.16 (0.85)	0.44**	0.49**	1.00	
4. Well-being	4.03 (0.64)	0.30**	0.42**	0.53**	1.00

*p < 0.05, **p < 0.01.

Correlation analyses were conducted to examine the relationships between these variables. Mindfulness was found to have a significant positive correlation with self-efficacy (*r* = 0.23*, *p* < 0.05) and self-regulation (*r* = 0.44**, *p* < 0.01). Self-efficacy and self-regulation were also positively correlated (*r* = 0.49**, *p* < 0.01). Furthermore, mindfulness, self-efficacy, and self-regulation exhibited significant positive correlations with psychological well-being, with correlation coefficients of 0.30**, 0.42**, and 0.53**, respectively, all at *p* < 0.01. These findings indicate that the study variables are interrelated, with significant positive correlations between mindfulness, self-efficacy, self-regulation, and psychological well-being, supporting the hypothesized relationships.

Furthermore, we probed for potential gender-related differences in our constructs by subjecting our data to four distinct independent-samples t-tests. These analytical efforts aimed to unearth any significant disparities between male and female participants in relation to the four core constructs. Notably, these rigorous tests revealed no statistically significant differences, underscoring the robustness and generalizability of our findings across gender groups.

Upon establishing the satisfactory fit of the measurement model, we proceeded to scrutinize various structural models, thereby putting our research hypotheses to the test. Initially, we conducted a comparative analysis between the hypothesized partial mediation model and the full mediation model. The latter involved setting all path coefficients from the predictor variables to the outcome variable to zero. In addition, we explored a rivaling direct model, in which all path coefficients to and from the mediator variable were constrained to zero.

Table 3 presents an examination of the fit indices pertaining to the alternate models, offering valuable insights into their capacity to elucidate the observed dataset. These indices are pivotal in assessing the models’ goodness of fit. As depicted in Table 3, the partial mediation model demonstrated superior fit indices [χ^2^ (432) = 7253.345, GFI = 0.859, CFI = 0.961, RMSEA = 0.044, TLI = 0.953, SRMSR = 0.062].

**Table 3:** Results of fit indices of alternative models.

Model	χ2	df	Δχ2	GFI	CFI	RMSEA	TLI	SRMR
Direct effect model	865.432**	440	–	0.832	0.912	0.068	0.901	0.182
Full mediation model	789.211**	436	76.221	0.841	0.943	0.051	0.928	0.071
Partial mediation model	723.345**	432	65.866	0.859	0.961	0.044	0.953	0.062

**p-value < 0.001, Δχ^2^, difference in chi-square values between the model and the subsequent model.

**Figure 1:**
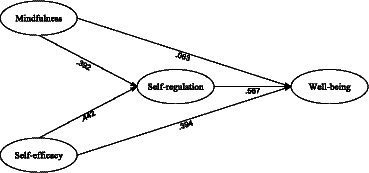
The partial mediation model.

Figure 1 illustrates the path diagram and parameter estimates for the partial mediation model, which exhibited a highly favorable fit concerning our dataset. Within Figure 1, it becomes evident that all path coefficients displayed statistical significance, with a single exception: the path linking mindfulness and psychological well-being did not attain statistical significance.

Table 4 displays the standardized path coefficients and t-values for the three structural models: the Direct Effects Model, the Full Mediation Model, and the Partial Mediation Model, analyzed using the Baron and Kenny (1986) method.

**Table 4:** Path estimates of structural model.

	Standardized path coefficients (*t*-value)		
	Direct model	Full model	Partial model
Self-efficacy → well-being	0.426 (6.19***)		0.394 (4.81**)
Mindfulness → well-being	0.136 (2.91*)		0.083 (1.09)
Self-efficacy → self-regulation		0.435 (6.13***)	0.442 (6.37***)
Mindfulness → self-regulation		0.356 (4.52**)	0.392 (4.80**)
Self-regulation → well-being		0.597 (7.48***)	0.567 (7.34***)

*p < 0.05, **p < 0.01, ***p < 0.001.

In the Direct Effects Model, several direct relationships were found to be statistically significant. The association between self-efficacy and well-being (*β* = 0.426, *t* = 6.19***) revealed a strong direct effect. Similarly, the link between self-efficacy and self-regulation (*β* = 0.435, *t* = 6.13***) was highly significant. The path from self-regulation to well-being (*β* = 0.597, *t* = 7.48***) also demonstrated a significant and robust relationship. In contrast, the relationship between mindfulness and well-being exhibited a smaller coefficient and was marginally significant (*β* = 0.136, *t* = 2.91*).

In the full mediation model, both the paths from self-efficacy to well-being and from mindfulness to well-being were mediated by self-regulation. In this model, the relationships between self-efficacy and self-regulation (*β* = 0.442, *t* = 6.37***) and between mindfulness and self-regulation (*β* = 0.392, *t* = 4.80**) were highly significant. These indirect paths clarified the connections between self-efficacy and mindfulness and well-being.

The partial model provided the best fit to the data. In this model, self-regulation mediated the relationships between mindfulness and well-being (*β* = 0.083, *t* = 1.09). In other words, mindfulness was related to well-being via the mediation of self-regulation (0.392 × 0.567 = 0.222). This suggests that while self-regulation plays a crucial role in explaining the relationship between these variables, there are additional factors contributing to the association between mindfulness and well-being.

Overall, the results from the Baron and Kenny (1986) method, as depicted in Table 4, indicate that the Partial Mediation Model offers the most suitable and comprehensive explanation of the relationships among self-efficacy, mindfulness, self-regulation, and well-being in this research framework.

## Discussion

The purpose of this study was to examine the interrelationships among mindfulness, self-efficacy, self-regulation, and their collective influence on the psychological well-being of individuals. In light of the growing significance of mental well-being in contemporary society, this research sought to unravel the intricate dynamics between these psychological constructs and shed light on how they jointly contribute to the overall psychological well-being of individuals, particularly in the context of EFL learners.

First, it was found that mindfulness directly correlates with the psychological well-being of EFL learners. This revelation harmonizes with an expanding body of research, further enriching our comprehension of how mindfulness practices can exert a positive influence on well-being within educational settings (e.g., Hoffman, 2010; Goretzki and Zysk, 2017; Lomas et al., 2017). The robust positive relationship between mindfulness and psychological well-being has been well-documented across diverse contexts, including educational environments (Chiesa and Serretti, 2009; Ager et al., 2015; Goretzki and Zysk, 2017; Zollars et al., 2019; Klussman et al., 2020). Our finding injects a fresh perspective by specifically underscoring the significance of mindfulness for EFL learners. These learners grapple with unique stressors, such as language barriers, cultural adaptation, and academic demands, all of which can profoundly impact their psychological well-being (Pan et al., 2023). By fostering self-awareness and emotional regulation, mindfulness practices offer EFL learners invaluable tools to navigate these challenges (Fathi et al., 2023).

The intricate connection between mindfulness and well-being in the domain of EFL learning is illuminated through a constellation of compelling mechanisms. Mindfulness practices, encompassing meditation and deep breathing exercises, have proven remarkably effective in assuaging stress and anxiety, common challenges encountered by EFL learners (Tang et al., 2015). For instance, mindfulness meditation’s ability to cultivate present-moment awareness and non-judgmental acceptance of thoughts and emotions effectively neutralizes the debilitating effects of stress, a significant concern for language learners navigating linguistic hurdles. Furthermore, mindfulness fosters self-compassion and self-acceptance, two cornerstones of psychological well-being. These qualities, nurtured by mindfulness’s cultivation of non-judgmental awareness, empower EFL learners to embrace their imperfections and navigate language learning challenges with resilience and composure (Neff, 2003; Hoffman, 2010; Ager et al., 2015; Lomas et al., 2017).

In addition, mindfulness’s transformative power extends beyond stress reduction and self-compassion, also bolstering cognitive and emotional resilience in the face of language learning challenges. This heightened resilience empowers EFL learners to approach language acquisition with unwavering determination and composure, enabling them to navigate setbacks and obstacles with resilience (Lomas et al., 2017; Fathi et al., 2023). Mindfulness cultivates a heightened state of attentiveness, sharpening the focus and concentration that are indispensable for effective language learning (Xue et al., 2019). This enhanced ability to concentrate allows EFL learners to immerse themselves fully in the linguistic landscape, absorbing grammatical nuances, vocabulary richness, and cultural subtleties with greater precision.

The heightened perceptiveness fostered by mindfulness extends beyond linguistic cues to encompass cultural nuances. Mindfulness allows EFL learners to appreciate the subtle cultural contexts surrounding language, enabling them to engage with the target language not as an isolated system but as an integral part of a vibrant cultural tapestry (Zeilhofer and Sasao, 2022). This deeper level of understanding fosters a more authentic and enriching language learning experience.

Second, our research unravels a vital revelation: self-efficacy directly associates with the psychological well-being of EFL learners. This revelation resonates with the existing body of knowledge that highlights the intricate interplay between self-efficacy and well-being (Siddiqui, 2015). The robust positive connection between self-efficacy and psychological well-being has been extensively documented in psychological research spanning a multitude of domains (Paciello et al., 2016; Phan, 2016; Cobo-Rendón et al., 2020; Etherton et al., 2022). Self-efficacy, as Bandura (1977) meticulously defined, refers to an individual’s belief in their capacity to perform tasks and surmount challenges. In the context of EFL learners, self-efficacy emerges as a potent psychological construct that profoundly shapes their language learning experiences (Hsieh and Kang, 2010). Learners with elevated self-efficacy tend to approach language acquisition with heightened motivation, unwavering persistence, and admirable resilience (Oxford, 1990; Mutlu et al., 2019). This, in turn, significantly contributes to elevated levels of psychological well-being (Cobo-Rendón et al., 2020).

The intricate relationship between self-efficacy and psychological well-being can be elucidated through various compelling mechanisms. EFL learners who harbor heightened self-efficacy are inherently inclined to set ambitious language-learning goals, propelled by their firm belief in their ability to attain them. The triumphant accomplishment of these challenging objectives invariably bestows upon them an augmented sense of competence and satisfaction, thus positively influencing their psychological well-being (Schunk and Pajares, 2005; Etherton et al., 2022).

Furthermore, self-efficacy plays an integral role in stress reduction and coping strategies (Piniel, 2013). EFL learners often encounter language-related stressors, including the apprehension of speaking in public or making errors. However, those endowed with high self-efficacy are better equipped to handle such stressors, driven by their unshakeable belief in their capacity to navigate these demanding situations. This, in turn, contributes to diminished levels of anxiety and fosters an elevated state of psychological well-being (Mills et al., 2006). Intriguingly, self-efficacious individuals exhibit a predilection for deploying more effective self-regulation strategies in their learning endeavors (Bouffard-Bouchard et al., 1991). This penchant for setting and achieving goals, monitoring their own learning progress, and adapting strategies as needed manifests as a sense of control over their linguistic journey (Yabukoshi, 2021; Rahimi and Fathi, 2022). Such mastery over self-regulation, in itself, is intrinsic to psychological well-being, as substantiated by the corpus of research in the field (Schunk, 1994; Zimmerman and Schunk, 2001).

In addition, our investigation brings to light a pivotal revelation: self-regulation acts as the linchpin in the intricate relationship between mindfulness and psychological well-being. Mindfulness, characterized by an unbiased awareness of the present moment, has been extensively linked to a spectrum of favorable outcomes for psychological well-being. These encompass a reduction in stress, anxiety, and depression (Hofmann et al., 2010), an upswing in life satisfaction (Brown and Ryan, 2003), and an enhancement in emotional regulation (Tang et al., 2015). However, the precise mechanisms through which mindfulness engenders these positive effects have garnered recent scholarly interest.

Our findings underscore the instrumental role of self-regulation as the mediator in translating mindfulness into augmented psychological well-being. Self-regulation, denoting an individual’s aptitude to govern and direct their thoughts, emotions, and behaviors, is actively fostered through mindfulness practices (Schunk, 1994). These practices cultivate heightened awareness of one’s thoughts and emotions, and furnish techniques to manage and channel them in a non-reactive, adaptive manner.

This finding is closely aligned with prior investigations that have demonstrated the augmentative impact of mindfulness practices on self-regulation (Leyland et al., 2019). For instance, mindfulness meditation has been shown to bolster attentional control and emotional regulation (Ostafin et al., 2015; Tang et al., 2015). By honing these self-regulation skills, individuals are better equipped to navigate stress, anxiety, and negative emotions, culminating in an overall enhancement of psychological well-being. This intrinsic connection between self-regulation and well-being is substantiated by a wealth of existing literature (e.g., Balkis and Duru, 2016; Gagnon et al., 2016; Fomina et al., 2020; Mascia et al., 2020).

Moreover, a seminal study by Chambers et al. (2008) unveils that self-regulation functions as the conduit through which mindfulness imparts its positive effects on well-being, even within clinical populations. This indicates that the mediating mechanism we observe extends its influence not only to general populations but also to those facing specific mental health challenges. These findings collectively fortify our result, further cementing the indispensable role of self-regulation in mediating the intricate relationship between mindfulness and psychological well-being.

Finally, our study unveils a critical pathway that elucidates the dynamic interplay between self-efficacy, self-regulation, and psychological well-being. At its core, self-efficacy, in accordance with Bandura’s seminal work (1977), signifies an individual’s conviction in their ability to not only perform tasks but also surmount challenges. This belief exerts a profound influence on motivation, goal-setting, and overall behavioral patterns (Bandura, 1989). When individuals possess high self-efficacy, they are more inclined to set ambitious goals, fueled by their unwavering belief in their capacity to attain them (Hawe et al., 2019). These proactive endeavors, in turn, serve as catalysts for an enhanced state of well-being (Schunk and Pajares, 2005; Mascia et al., 2023).

Central to this nexus is the pivotal role played by self-regulation, defined as an individual’s capacity to govern and orchestrate their thoughts, emotions, and behaviors. Those endowed with high self-efficacy demonstrate a remarkable aptitude for effective self-regulation, driven by their unshakable belief in their capabilities (Feldmann et al., 1995). This dynamic self-regulation process becomes a linchpin, contributing significantly to the augmentation of psychological well-being (Gagnon et al., 2016; Mascia et al., 2020).

Our findings harmonize with previous research, echoing the sentiment that self-regulation is a linchpin in goal pursuit and personal growth (Zimmerman and Schunk, 2001; Dignath et al., 2008). Individuals with heightened self-efficacy demonstrate superior self-regulation skills, adept at setting and monitoring their progress toward their objectives. This mastery over self-regulation not only affords them a heightened sense of control but also fosters a pervasive feeling of competence, thereby positively impacting psychological well-being (Balkis and Duru, 2016; Gagnon et al., 2016; Fomina et al., 2020). This illuminates the expansive reach of self-efficacy, not only in motivating and goal-setting, but also in influencing an individual’s capacity for self-regulation (Trautner and Schwinger, 2020). Our findings extend this intricate relationship to the realm of psychological well-being, providing further validation for the indispensable mediating role of self-regulation in this intricate interplay. In essence, our study reaffirms and amplifies the understanding of how self-efficacy, underpinned by robust self-regulation, contributes significantly to the cultivation and sustenance of psychological well-being.

## Conclusion

In summary, our research has revealed the intricate interplay between self-efficacy, self-regulation, and psychological well-being. We have shown that self-efficacy significantly influences one’s psychological well-being, with self-regulation acting as a vital mediator in this relationship. Individuals with high self-efficacy tend to demonstrate enhanced self-regulation skills, which, in turn, contribute to their improved mental health. This discovery aligns with existing literature on self-efficacy and self-regulation, further highlighting their importance in fostering psychological well-being. Our study underscores the critical need to promote self-efficacy and self-regulation in various contexts, including education, clinical psychology, and personal development efforts. Interventions aimed at enhancing these psychological constructs can have profound effects on individuals’ overall mental health. By recognizing the mediating role of self-regulation, we have provided a more comprehensive understanding of the pathways through which self-efficacy impacts psychological well-being. Ultimately, our research highlights the potential for targeted interventions and strategies to enhance self-efficacy and self-regulation, providing individuals with the means not only to achieve their goals but also to enhance their psychological well-being. This knowledge constitutes a valuable contribution to the broader field of psychology and personal development.

The implications of our findings extend to various domains, spanning education, clinical psychology, and individual well-being enhancement. Educators are empowered to play a crucial role in nurturing self-efficacy and self-regulation among students. They can employ strategies that encourage goal setting, provide constructive feedback, and promote self-monitoring to foster self-efficacy. Simultaneously, the development of self-regulation skills can be seamlessly integrated into teaching methodologies, equipping students with the tools to manage their learning effectively. This, in turn, can lead to improved psychological well-being and academic success.

Furthermore, in the realm of clinical psychology, therapists and mental health practitioners are poised to enhance their interventions by incorporating self-efficacy-building activities. By recognizing the mediating role of self-regulation in the relationship between self-efficacy and psychological well-being, therapists can tailor their approaches to enhance both self-efficacy and self-regulation in their clients. This approach can result in more comprehensive and effective treatments for psychological well-being. For individuals seeking to improve their psychological well-being, our findings offer valuable guidance. By focusing on self-efficacy and self-regulation enhancement, individuals can engage in activities that challenge and expand their self-efficacy beliefs, set achievable goals, and practice self-regulation strategies. This approach can have a positive impact on their mental health and overall well-being.

The findings of our study contribute to a growing body of research exploring the interrelationships between self-efficacy, self-regulation, and psychological well-being in EFL learners. However, several limitations warrant consideration to further enhance the study’s depth and generalizability. Firstly, the cross-sectional design of our study, which involved collecting data at a single point in time, restricts our ability to establish causal relationships between variables and to fully capture the dynamic nature of the constructs examined. Longitudinal studies that track participants over time would provide a more nuanced understanding of how these constructs evolve and interact with each other over an extended period.

Secondly, the reliance on self-report measures, while insightful, may introduce biases or inaccuracies due to social desirability or memory limitations. To strengthen the validity of our findings, future research could consider complementing self-reports with objective measures, such as behavioral observations or physiological data, which would provide independent assessments of the constructs under investigation. Thirdly, the limited sample representation, consisting of EFL learners from a single university in China, raises concerns about the generalizability of our findings to broader populations with diverse cultural backgrounds, educational levels, and socioeconomic backgrounds. Expanding the sample and ensuring representation from a wider range of individuals would enhance the applicability of our results to a broader spectrum of EFL learners.

In addition, while our study provided evidence for the mediating role of self-regulation in the relationship between self-efficacy and psychological well-being, further exploration of the underlying mechanisms is needed. Delving deeper into the precise processes by which self-regulation fosters psychological well-being among EFL learners would provide a more comprehensive explanation for the observed associations. Finally, the absence of control variables in our study, such as personality traits, coping strategies, and life stressors, could have influenced the relationships between the constructs examined. Incorporating these control variables in future research would allow for a more rigorous examination of the direct and indirect effects of self-efficacy, self-regulation, and psychological well-being on each other.

In summary, our research contributes to the understanding of the intricate interplay between self-efficacy, self-regulation, and psychological well-being. We emphasize the need for interventions promoting self-efficacy and self-regulation in various contexts, including education, clinical psychology, and personal development. Educators, therapists, and individuals seeking to enhance their well-being can benefit from our findings, which provide valuable guidance for incorporating strategies that challenge and expand self-efficacy beliefs and foster effective self-regulation. While our study offers valuable insights, it is crucial to acknowledge its limitations, such as the cross-sectional design and reliance on self-report measures. Future research should explore the dynamic nature of these constructs longitudinally and consider diverse samples for broader generalizability. Additionally, further investigations into the specific mechanisms underlying the mediation of self-regulation in the relationship between self-efficacy and psychological well-being are warranted.

## Data availability statement

The data analyzed in this study is subject to the following licenses/restrictions: the raw data supporting the conclusions of this article will be made available by the authors, without undue reservation. Requests to access these datasets should be directed to LF, fanlijuanwfust@163.com.

## Ethics statement

The studies involving humans were approved by College of Teacher Education, Weifang University of Science and Technology, Weifang 262700, China. The studies were conducted in accordance with the local legislation and institutional requirements. The participants provided their written informed consent to participate in this study.

## Author contributions

LF: Conceptualization, Data curation, Formal analysis, Funding acquisition, Investigation, Methodology, Project administration, Resources, Software, Supervision, Validation, Visualization, Writing – original draft, Writing – review & editing. FC: Conceptualization, Data curation, Formal analysis, Funding acquisition, Investigation, Project administration, Resources, Software, Supervision, Visualization, Writing – original draft, Writing – review & editing.
